# A Multidimensional Assessment of Food Security in Low- and Middle-Income Countries: System Performance and Interdimensional Coordination

**DOI:** 10.3390/nu18091432

**Published:** 2026-04-30

**Authors:** Na Li, Xinyi Song, Mengze Liu, Yang Hao, Jiajun Liu, Zuokun Liu, Yuyang Zhang, Minmin Wang, Minghui Ren

**Affiliations:** 1 Department of Global Health, School of Public Health, Peking University, Beijing 100191, China; lina2021@bjmu.edu.cn (N.L.);; 2 China Center for Health Development Studies, Peking University, Beijing 100191, China; 3 Beijing Institute for Health Development, Peking University, Beijing 100191, China; 4 National Health Commission Key Laboratory of Health System Reform and Governance (Peking University), Beijing 100191, China; 5 Institute for Global Health, Peking University, Beijing 100191, China

**Keywords:** food security, low- and middle-income countries, entropy-weighted TOPSIS, coupling coordination degree, One Health

## Abstract

Background: Food security systems are central to nutritional health and Sustainable Development Goal 2 (SDG 2), yet existing assessments have paid limited attention to cross-dimensional coordination within food security systems. This study assessed both system performance and coordination in low- and middle-income countries (LMICs) during 2019–2021. Methods: Based on a multidimensional 25-indicator framework, the entropy-weighted Technique for Order Preference by Similarity to an Ideal Solution (TOPSIS) approach was used to evaluate system performance. Spearman’s rank correlation and Bland–Altman agreement analyses against the SDG 2 Index and the Under-Five Mortality Rate (U5MR) were used to examine the validity. The coupling coordination degree (CCD) model was used to assess coordination across the four dimensions of food security: availability, access, utilization, and stability. Results: Among all included LMICs, composite scores ranged from 0.103 to 0.698. Regionally, Europe and Central Asia showed the strongest overall performance (mean = 0.54), whereas Sub-Saharan Africa exhibited the lowest levels (mean = 0.27). The dimensions of access and stability were identified as the principal global bottlenecks of overall food security system development. The proposed index correlated positively with the SDG 2 Index (*R* = 0.662, *p* < 0.001) and inversely with the U5MR (*R* = −0.769, *p* < 0.001). The coupling degrees were consistently high but exceeded coordination levels across regions, indicating that strong interdependence among dimensions did not necessarily translate into balanced or synergistic system development. Conclusions: Food security systems in LMICs are constrained by weaknesses in the access and stability dimensions, as well as by insufficient cross-dimensional coordination. Strengthening them requires integrated, cross-sectoral strategies that enhance both system performance and interdimensional coordination.

## 1. Introduction

Ensuring global food security is not merely a target of Sustainable Development Goal 2 (SDG 2)—Zero Hunger—but a foundational pillar of global health security and the “One Health” strategy [1,2,3]. In an era characterized by accelerating climate change and emerging infectious threats, the food system functions as a foundational determinant of global health security and health resilience [4]. A well-development food system is essential for buffering populations against climate-sensitive health risks by ensuring the nutritional stability required for disease resistance and recovery. Conversely, systemic deficiencies in food security systems compromise the capacity of public health systems to respond to crises, creating a syndemic environment where malnutrition, environmental stressors, vulnerability and susceptibility mutually reinforce one another [5,6]. Therefore, evaluating food security requires a paradigm shift: from a static metric of caloric availability to a multidimensional assessment of a system’s structural capacity to support “One Health” objectives and mitigate compounded health burdens.

According to the Food and Agriculture Organization (FAO) of the United Nations, the leading authority on global food security, the conceptual framework of food security was built upon four interconnected dimensions: availability, access, utilization, and stability [7]. Food availability refers to the physical supply of food through production, stock levels, and trade, ensuring sufficient quantities are present; access relates to whether individuals and households have the economic and physical means to acquire the available food; utilization deals with the body’s ability to absorb and metabolize nutrients from food, dependent on food safety, hygiene, and health; and stability ensures that the other three dimensions are consistently maintained over time, guarding against shocks and crises like economic downturns or natural disasters [8]. This framework helps to systematically understand the complexities of food security and provides a comprehensive blueprint for analysis and policymaking. In view of the deep interconnection among these four dimensions, deficiencies in one area undermine progress in the others, and focusing on a single dimension in isolation often fails to address interrelated challenges. However, the current assessment of food security systems exhibits a structural imbalance that obscures this systemic complexity. Moreover, the critical trade-offs and synergies between different dimensions are often neglected [9,10]. A systematic analysis of 78 studies shows that household-level calorie adequacy is the most frequently used sole measure (22%), followed by dietary diversity-based (44%) and experience-based (40%) indicators. In contrast, the utilization (13%) and stability (18%) dimensions are seldom captured, and only three of the retrieved publications employed a comprehensive food security measurement by considering all four food security dimensions [11,12,13,14]. In addition, existing models often overlook underlying determinants shared with the health sector, such as Water, Sanitation, and Hygiene (WASH) infrastructure, anthropometry measures and political stability, which are respectively important components of the utilization and stability dimensions [11]. This oversight hinders a holistic understanding of how food systems interact with broader health determinants, leaving policymakers blind to the specific bottlenecks that undermine both nutritional security and disease prevention. Beyond these dimensional gaps, the methodological rigor of food security assessment requires advancement. Previous assessment typically relies on linear aggregation, like weighted sums, which assumes that high performance in one dimension can compensate for failure in another [15,16]. This assumption is flawed in the context of system theory. A high aggregate score may mask severe internal imbalances, for instance, high agricultural output (availability) coexisting with fragile supply chains (access) or poor sanitary environments (utilization). Such dysfunctional high performance creates a fragile system unable to withstand climate shocks or health emergencies. Consequently, there is a pressing need to move beyond calculating development levels to quantifying the coupling coordination degree (CCD), a metric that evaluates the internal synergy, structural balance, and interactive coherence among the four dimensions [17,18].

To address these challenges, this descriptive study establishes a dual-perspective assessment framework for low- and middle-income countries (LMICs), utilizing a comprehensive 25-indicator system developed by the FAO. Combining objective performance evaluation of food security systems with diagnosis of the structural harmony of the system, this approach allows us to distinguish between resilient, balanced systems and those that are outwardly high-scoring but internally disjointed. Furthermore, to ensure the findings bear practical relevance for health policy, we validate the results against two critical benchmarks: the SDG 2 Index and the Under-5 Mortality Rate (U5MR). By integrating objective weighting, structural coordination analysis, and validation against public health indicators, this research aims to identify systemic levers for enhancing the co-benefits of food security and health systems, thereby supporting the implementation of One Health strategies in the face of climate change.

## 2. Methods

### 2.1. Indicator System and Data Collection

The FAO presented a set of high-quality and internationally comparable indicators, which have internationally recognized definitions and standards [19]. As the UN is regarded as a trustworthy organization that has considered the comparability and reliability of data sources and indicators, its data sources and original indicator system of food security are adopted in this study. The original dataset used in this study was downloaded as a table in csv format from the Suite of Food Security Indicators published in the FAO Statistics database [19]. Based on the World Bank country classifications by income level published in 2021, this study included all countries that belonged to the low-income, lower-middle-income, and upper-middle-income categories and for which data were available in the FAO database.

### 2.2. Indicator Direction Identification

**Figure 1:**
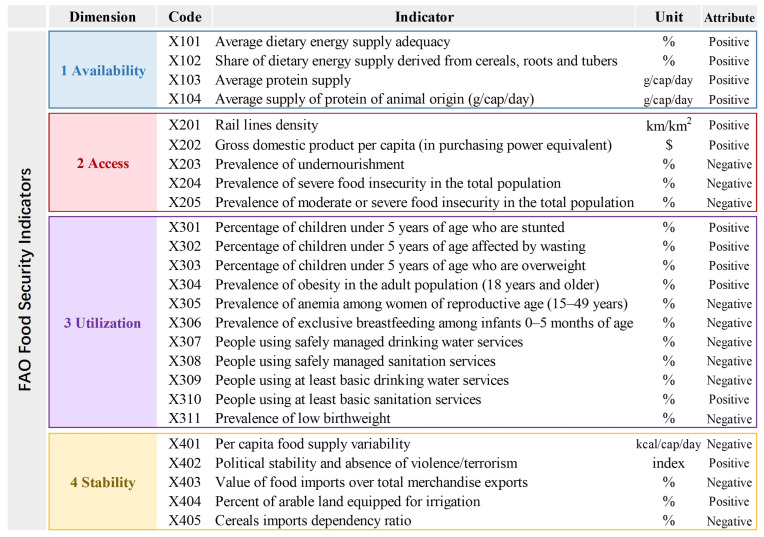
Food Security Indicators proposed by Food and Agriculture Organization.

In the entropy-weighted TOPSIS method, it is necessary to normalize all indicators into a uniform type to eliminate the influence of different indicator directions. In this study, all indicators were converted into a positive type, meaning that larger values represent better food security performance. Accordingly, the indicators within each dimension are classified as positive and negative before transformation. A positive indicator implies that a larger value corresponds to a higher level of food security; conversely, a negative indicator implies that a larger value corresponds to a lower level of food security. The detailed statistical concepts, definitions, and relevance of each indicator to its respective dimension are available on the website of the FAO Statistics database. These directional relationships determined the formulas applied for standard homogenization (see Figure 1 for all indicators).

### 2.3. Weighting and Evaluation Methods

We adopted the Entropy Weight Method (EWM) and the Technique for Order of Preference by Similarity to Ideal Solution (TOPSIS) to evaluate the performance of the national food security system. The EWM–TOPSIS is a method that uses the EWM to determine the index weight and calculates the proximity degree to determine the importance degree. In information theory, entropy measures uncertainty and quantifies an attribute’s dispersion. Smaller entropy indicates greater dispersion, more information, and higher weight in comprehensive evaluation, while the inverse relationship holds for larger entropy. Equal attribute values yield zero entropy, rendering the attribute irrelevant. Thus, entropy enables weight calculation for multi-attribute comprehensive evaluation [20]. This method has the advantages of making full use of the original data with little loss of information and calculating objective and scientific results [20,21]. Compared with subjective weighting tools such as the analytic hierarchy process and the Delphi method, the EWM determines the weight according to the degree of change in the original data, thereby providing a more objective weighting of indicators. The TOPSIS is a common approach for solving multi-objective decision-making problems using the ideal method [22,23,24]. Furthermore, EWM offers significant advantages over the average weighting method (equal weights for each index) used in TOPSIS. The calculation steps of the EWM-TOPSIS method are as follows:

Step 1. Suppose there are *M* evaluation objects, *M* = *M*_1_, *M*_2_, …, *M_m_*, and each evaluation object has *N* evaluation indicators: *N* = *N*_1_, *N*_2_, …, *N_n_*. Then the matrix can be established:

$$X=\left[\begin{array}{cccc} x_{11} & x_{12} & \ldots & x_{1m}\\ x_{21} & x_{22} & \ldots & x_{2m}\\ \vdots & \vdots & \ddots & \vdots \\ x_{n1} & x_{n2} & \ldots & x_{nm} \end{array}\right]$$

Step 2. Initial processing of data. Due to the different meanings, units, and orders of magnitude of each indicator, the indicators cannot be directly calculated. Therefore, to standardize the original data, we perform the following calculations:

$$\begin{array}{c} \mathrm{For}\;\mathrm{positive}\;\mathrm{indicators}:\;z_{ij}=\frac{x_{ij}-x_{j}^{\min}}{x_{j}^{\max}-x_{j}^{\min}},\\ \mathrm{For}\;\mathrm{negative}\;\mathrm{indicators}:\;z_{ij}=\frac{x_{j}^{\max}-x_{ij}}{x_{j}^{\max}-x_{j}^{\min}},\\ \mathrm{For}\;\mathrm{moderate}\;\mathrm{indicators}:\;z_{ij}=\left\{\begin{array}{cc} 1-\frac{a-x_{ij}}{\max\left(a-x_{j}^{\min},x_{j}^{\max}-b\right)} & ,x_{ij}<a\\ 1 & ,a\leq x_{ij}\leq b\\ 1-\frac{x_{ij}-b}{\max\left(a-x_{j}^{\min},x_{j}^{\max}-b\right)} & ,x_{ij}>b \end{array}\right. \end{array}$$

Step 3. Calculate the characteristic proportion of each indicator in each evaluation object.

The normalized matrix at this time *Z* is as follows:

$$Z=\left[\begin{array}{cccc} z_{11} & z_{12} & \ldots & z_{1m}\\ z_{21} & z_{22} & \ldots & z_{2m}\\ \vdots & \vdots & \ddots & \vdots \\ z_{n1} & z_{n2} & \ldots & z_{nm} \end{array}\right]$$

Then the proportion of object *i* under indicator *j* to the total of that indicator is denoted as *P_ij_*:

$$P_{ij}=z_{ij}/\sum_{i=1}^{n}z_{ij},(j=1,2,\cdots ,m)$$

Step 4. Calculate the entropy value of the evaluation indicators:

$$E_{j}=-\frac{1}{\ln n}\sum_{i=1}^{n}P_{ij}lnP_{ij},(j=1,2,\cdots ,m)$$

Step 5. Calculate the coefficient of variation (CV) of the evaluation indicators *G_j_*:

$$G_{j}=1-E_{j}$$

Step 6. Determine the entropy weight of the evaluation indicators *Wj*:

$$W_{j}=G_{j}/\sum_{j=1}^{m}G_{j}$$

Step 7. Combine the entropy weight to construct the weighted standardized matrix *Z*:

$$Z^{*}=\left[\begin{array}{cccc} \omega_{1}\times z_{11} & \omega_{2}\times z_{12} & \ldots & \omega_{m}\times z_{1m}\\ \omega_{1}\times z_{21} & \omega_{2}\times z_{22} & \ldots & \omega_{m}\times z_{2m}\\ \vdots & \vdots & \ddots & \vdots \\ \omega_{1}\times z_{n1} & \omega_{2}\times z_{n2} & \ldots & \omega_{m}\times z_{nm} \end{array}\right]$$

Step 8. Determine the ideal solution. Let *Z*^+^ be the most preferred option (positive ideal solution) and *Z*^−^ be the least preferred option (negative ideal solution):

$$Z^{+}=(Z_{1}^{+},Z_{2}^{+},\cdots ,Z_{m}^{+})$$

$$Z^{-}=(Z_{1}^{-},Z_{2}^{-},\cdots ,Z_{m}^{-})$$

Step 9. Calculate the distance between the evaluation objects and both the positive ideal solution and the negative ideal solution:

$$D_{i}^{+}=\sqrt{\sum_{j=1}^{m}{\left(Z_{j}^{+}-z_{ij}^{*}\right)}^{2}}$$

$$D_{i}^{-}=\sqrt{\sum_{j=1}^{m}{\left(Z_{j}^{-}-z_{ij}^{*}\right)}^{2}}$$

Step 10. Calculate the closeness of the evaluation object to the positive ideal solution:

$$S_{i}=\frac{D_{i}^{-}}{D_{i}^{+}+D_{i}^{-}}$$

The current study uses closeness as the comprehensive evaluation score of each object. Closeness ranges from 0 to 1. The larger it is, the farther it is from the negative ideal solution, and it also indicates that the comprehensive evaluation score of the object is higher.

The four dimensions of food security can be considered four subsystems within the overall food security system. In this study, the entropy-weighted TOPSIS method was first employed to calculate the weights of the indicators within each dimension, from which dimension-specific scores were derived. A composite score encompassing all four dimensions was then computed. Given their equal importance to the integrated development of the system, each dimension was assigned a weight of 0.25. Based on this weighting scheme, the comprehensive weights of all 25 indicators within the food security system were calculated. It should be noted that all LMICs were included in this study. However, since the total food security score and the coupling coordination degree analysis require valid scores for all four dimensions, no missing values are permitted for indicators under any dimension. Countries with missing data were excluded from the calculation of the composite score and the coupling coordination degree model. The weights of the indicators and dimension-specific score results are provided in the Supplementary Materials.

### 2.4. Statistical Validation Strategy

To evaluate the criterion validity and empirical relevance of the proposed multidimensional food security index, we conducted an external validation using two globally recognized benchmarks: the SDG 2 score and the U5MR. The selection of these benchmarks was grounded in their authoritative standing and their conceptual alignment with the multidimensional nature of food security. Firstly, the SDG 2 score was utilized as a policy-oriented benchmark. As the United Nations’ primary metric for monitoring progress toward “Zero Hunger,” the SDG 2 score represents the global consensus on food security governance. By correlating our index with this benchmark, we aimed to assess the criterion validity of our model—specifically, whether our objective weighting approach aligns with the internationally recognized progress of nations in eradicating hunger and malnutrition. Secondly, the U5MR was selected as a distal health-outcome benchmark. The U5MR is widely acknowledged as one of the most sensitive indicators of socioeconomic development and population health in the context of LMICs. Since food insecurity is a fundamental driver of child mortality, the U5MR serves as a critical yardstick for concurrent validity. Validating the index against the U5MR ensures that the evaluation framework is not merely a theoretical construct but a meaningful reflection of the actual public health burden associated with food system vulnerabilities.

A dual-step statistical approach was employed to evaluate the validity of the FSI. First, Spearman’s rank correlation coefficients were calculated to quantify the strength and direction of the correlations. Scatter plots with fitted linear regression lines were generated to visually assess the overall trends. However, as high correlation may not necessarily imply good agreement, Bland–Altman analysis was subsequently conducted to assess the quantitative interchangeability of the metrics [25,26]. This method allowed for the visualization of the differences between the FSI and the benchmarks against their means, facilitating the identification of any systematic bias (mean difference) and the determination of the 95% Limits of Agreement (LoAs).

### 2.5. Coupling Coordination Analysis of Food Security Systems

The coupling coordination degree model (CCD model) was used to diagnose the structural equilibrium and internal synergy among the four dimensions. While the aggregate score reflects the weighted average performance, it may mask systemic vulnerabilities where a single lagging dimension compromises the whole. These four dimensions can be regarded as four subsystems of the whole food security system. The CCD model, as an available tool for identifying multivariate coupling relationships among different systems, can demonstrate the synergistic result and overall effectiveness of the coupled system [27,28]. The CCD analysis decomposes this relationship into coupling degree (C-value), reflecting the strength of interactions or dependencies, and coupling coordination degree (D-value), reflecting the level of cooperative development [27,29]. The CCD model is given in the formula

$$C={\left(\frac{\prod_{i=1}^{n}U_{i}}{{\left(\frac{1}{n}\sum_{i=1}^{n}U_{i}\right)}^{n}}\right)}^{\frac{1}{n}},$$

where *n* represents the number of subsystems, and *U_i_* denotes the score of each subsystem. A larger *C*-value indicates smaller dispersion among subsystems, stronger interactions, and a higher coupling degree. In this study, *n* = 4, and the four dimensions are considered four subsystems.

$$\begin{array}{c} T=\alpha_{1}\times U_{1}+\alpha_{2}\times U_{2}+\alpha_{3}\times U_{3}+\alpha_{4}\times U_{4}\\ D=\sqrt{C\times T} \end{array}$$

Here, α denotes the weight of each dimension, and $U_{1-4}$ represents the score of each dimension. As each dimension is considered equally important, $\alpha_{1}$ = $\alpha_{2}$ = $\alpha_{3}$ = $\alpha_{4}$ = 0.25.

## 3. Results

### 3.1. Multidimensional Assessment of Food Security Performance

**Figure 2:**
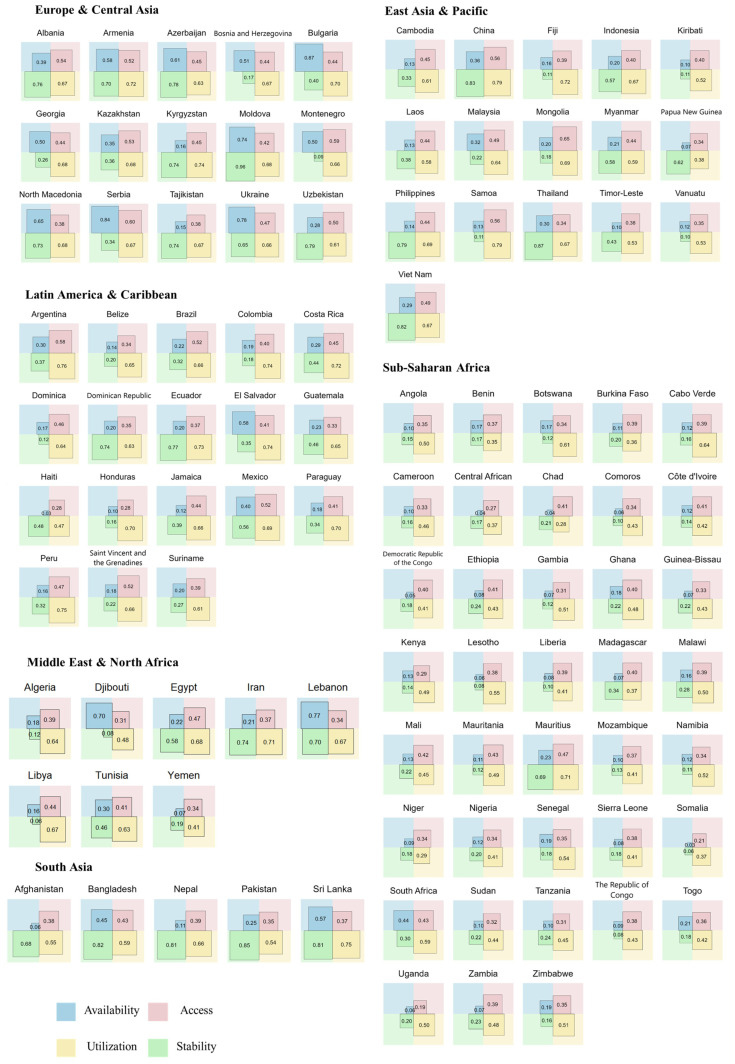
Dimension-specific scores of food security system across LMICs during 2019–2021.

The application of the entropy-weighted TOPSIS method revealed substantial heterogeneity in food security performance across the 131 LMICs analyzed. A total of 100 countries with complete data across all variables were included in the final multidimensional assessment. The comprehensive FSI scores exhibited a wide dispersion, ranging from a minimum of 0.103 (Eritrea) to a maximum of 0.698 (Moldova), underscoring severe food security development disparities at the national level (Figure 2 and Table S2 in Supplementary Material). High-performing nations were predominantly concentrated in the Europe and Central Asia region, with Moldova (0.698), Ukraine (0.638), and China (0.634) ranking among the top tier. Conversely, the lowest quartile was dominated by countries in Sub-Saharan Africa and the Middle East and North Africa, with critical deficits observed in Burundi (0.129), Equatorial Guinea (0.125), and Somalia (0.166). Notably, a high aggregate score did not necessarily imply balanced development across all domains. For instance, while China demonstrated exceptional performance in utilization (0.791) and stability (0.834), its scores in availability (0.557) and access (0.356) were relatively moderate, highlighting specific structural bottlenecks despite a high overall ranking. Similarly, Pakistan exhibited a high stability score (0.853) but lagged significantly in the access dimension (0.251), reflecting internal systemic imbalances.

#### Dimension-Specific Performance

The results of single-dimensional analysis revealed that the stability dimension exhibited the highest variation globally, with scores ranging from 0.062 (Libya) to 0.955 (Moldova). The access dimension represented a global vulnerability, with universally low scores across most developing regions, particularly in nations like Haiti (0.031) and Afghanistan (0.057). Conversely, the utilization dimension showed the highest consistency and relatively higher average scores (global mean = 0.61). Data availability varied across dimensions. Missing values, indicated by “–” in the corresponding table, were observed for the access dimension in nine countries (e.g., Belarus, Turkey, Turkmenistan) and for the stability dimension in seven countries (e.g., Micronesia, Solomon Islands). These gaps may affect the accuracy of the composite scores for the affected countries (Supplementary Material).

### 3.2. Regional Patterns and Dimensional Disparities

**Figure 3:**
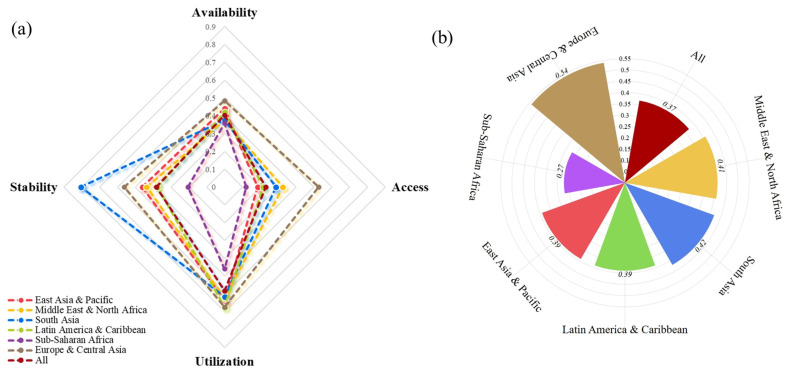
Dimension-specific (**a**) and comprehensive (**b**) scores of the food security system by geographic region during 2019–2021.

At the regional level, the analysis identified a distinct hierarchical structure in food security performance (Figure 3). Europe and Central Asia emerged as the highest-performing region, with a mean comprehensive score of approximately 0.54, driven by robust scores across all four dimensions. In contrast, Sub-Saharan Africa presented the most precarious profile, recording the lowest regional average (0.27). This region is characterized by systemic weaknesses across the board, particularly in availability and access, as visualized by the constricted inner polygon in the radar chart (Figure 3a). The remaining regions, East Asia and Pacific (0.42), Middle East and North Africa (0.41), Latin America and the Caribbean (0.39), and South Asia (0.39), occupied an intermediate tier, yet their underlying dimensional configurations varied significantly. South Asia displayed a unique “high-stability, low-access” profile. While it achieved high scores in the stability dimension (blue dotted line in Figure 3a), its performance in access was comparable to that of Sub-Saharan Africa, suggesting that while food supply might be stable over time, economic or physical access remains a critical barrier. Latin America and the Caribbean and the Middle East and North Africa showed relatively balanced but mediocre performance, with no single dimension standing out as exceptionally strong or weak compared to the global average.

### 3.3. Validation Against External Benchmarks

**Figure 4:**
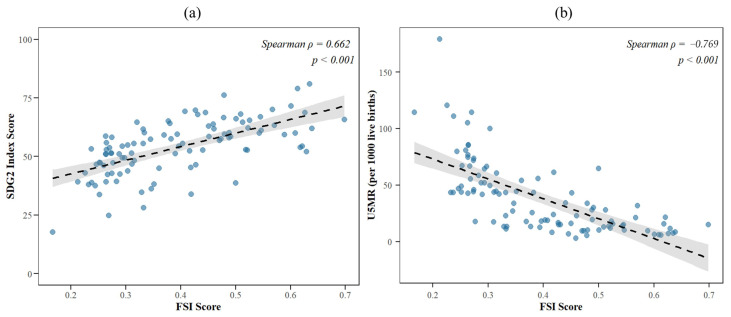
Spearman’s rank correlation analysis of the food security index score against external validation benchmarks. Notes: Panels illustrate the association between the FSI score and (**a**) the SDG 2 Index Score and (**b**) the Under-5 Mortality Rate (U5MR). The dashed black lines represent the fitted linear regression trends, while the shaded gray areas indicate the 95% confidence intervals. The Spearman correlation coefficients and *p*-values are annotated in the upper right corner of each plot.

The criterion validity of the proposed Multidimensional FSI was assessed using Spearman’s rank correlation analysis against two established indicators: the SDG 2 Index and the Under-5 Mortality Rate (U5MR). Figure 4 illustrates the scatter plots with superimposed linear regression trend lines. The analysis revealed a statistically significant positive correlation between the FSI and the SDG 2 Index (*R* = 0.662, *p* < 0.001), indicating a moderate-to-strong consistency between the proposed index and the global zero-hunger targets. Conversely, a strong significant inverse association was observed between the FSI and U5MR (*R* = −0.769, *p* < 0.001). This negative correlation confirms that higher FSI scores are robustly associated with lower child mortality burdens in the studied countries.

**Figure 5:**
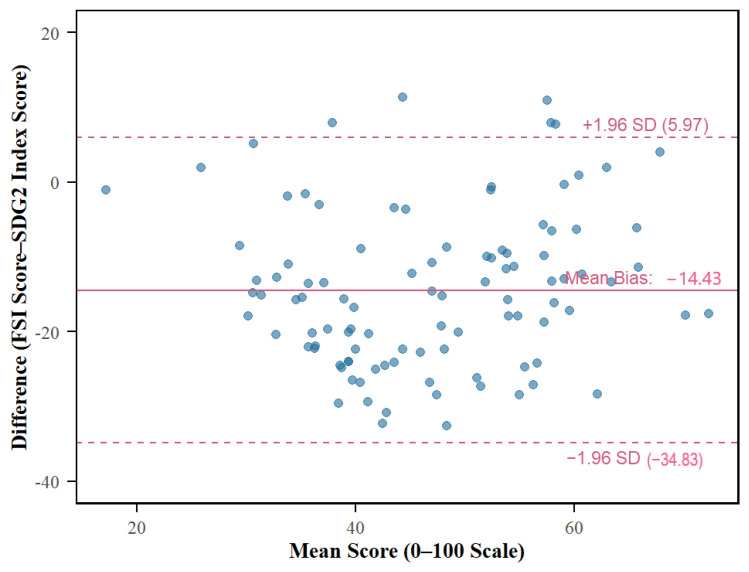
Bland–Altman plots assessing the methodological agreement between the multidimensional food security index (FSI) scores and external benchmarks.

Figure 5 presents the Bland–Altman plots quantifying the agreement between the FSI and external indicators. When compared against the SDG 2 Index (scaled 0–100), a significant mean difference of −14.43 was observed (Figure 5). This negative bias implies that the FSI is calibrated more conservatively than the SDG 2 Index, likely due to its incorporation of broader structural constraints. Despite this systematic shift, the distribution of differences remains stable, indicating that the two indices measure a conceptually similar construct. This confirms that the FSI possesses a high degree of construct validity when benchmarked against key health outcomes.

The figure displays the agreement with the SDG 2 Index Score (scaled 0–100), revealing a systematic negative bias. The solid red lines represent the mean difference (bias), while the dashed red lines indicate the 95% Limits of Agreement (Mean ± 1.96 SD). Specific bias values and LoA ranges are annotated within the plots.

### 3.4. Coupling Coordination Degree of Food Security Dimensions

**Figure 6:**
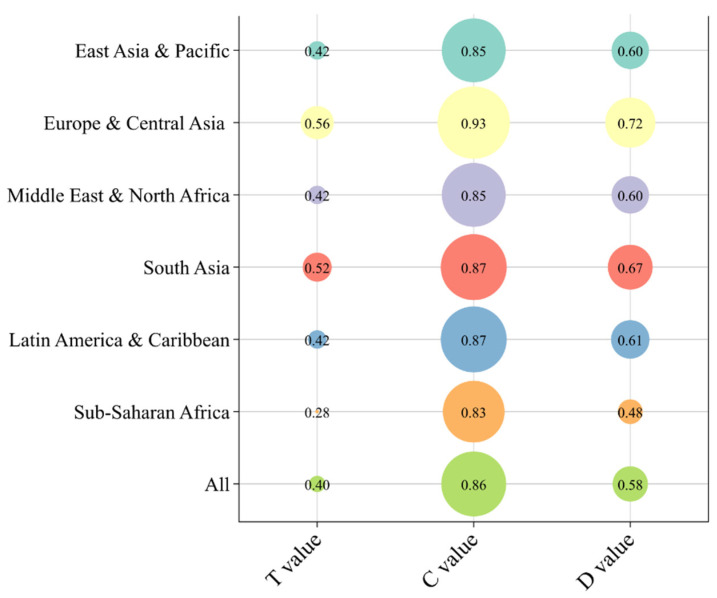
The coupling coordination degree among four dimensions in food security by geographic region during 2019–2021.

Figure 6 presents the results for the coupling degree (C-value), comprehensive development index (T-value), and coupling coordination degree (D-value) across the studied regions. The study found consistent disparity between the coupling degree (C-value) and the coupling coordination degree (D-value) across all investigated regions. The global mean for LMICs exhibited a higher C-value relative to the D-value. The coupling degree (C-value) remained consistently high across all regions with values exceeding 0.80, confirming that the four pillars of food security function as a highly interdependent organic whole rather than isolated components. However, the coordination degree (D-value) exhibits heterogeneity, ranging from 0.48 to 0.72. Such coordination lag indicates that while the dimensions are tightly linked, they do not always develop in a synchronized or mutually reinforcing manner. Specifically, Europe and Central Asia exhibited the highest level of coordination (D = 0.72), falling within the classification of intermediate coordination. This region also recorded the highest values for both coupling intensity (C = 0.93) and comprehensive development (T = 0.56). South Asia (D = 0.67) and Latin America and the Caribbean (D = 0.61) were classified under primary coordination. Both regions maintained high coupling degrees (C = 0.87) but showed moderate comprehensive development scores relative to Europe and Central Asia. East Asia and Pacific and the Middle East and North Africa both recorded a D-value of 0.60, with coupling degrees of approximately 0.86 and comprehensive development scores of 0.42. Sub-Saharan Africa recorded the lowest coordination level (D = 0.48). While the region’s coupling degree remained high (C = 0.83), its comprehensive development index was the lowest among all regions (T = 0.28).

The bubble chart displays the coupling coordination degree (D-value), coupling degree (C-value), and composite development degree (T-value) across regions. The size of the bubbles represents the magnitude of the values, highlighting that high coupling does not guarantee high coordination without sufficient coordination.

## 4. Discussion

This study used an integrated assessment framework combining the entropy-weighted TOPSIS and CCD models to systematically evaluate the multidimensional food security of 131 LMICs. First, the entropy-weighted TOPSIS assessment, conducted at both dimensional and comprehensive levels, reveals spatial differences in food security development among LMICs. The analysis reveals regional disparities, with Europe and Central Asia demonstrating stronger performance, while Sub-Saharan Africa and the Middle East and North Africa regions lag behind. The dimensional breakdown further identifies persistent structural constraints hidden within these aggregate scores globally. Although the scores of the utilization dimension were relatively higher, the access and stability dimensions remained consistently low in most developing regions. This structural deficit identifies economic accessibility and systemic resilience as the key barriers to the overall food security system. These quantified results not only locate each country within the global food security landscape but also highlight the uneven developmental trajectories that traditional single-indicator assessments often obscure. Second, the convergent validity of the proposed FSI was confirmed by its strong correlation and methodological agreement (Bland–Altman analysis) with the SDG 2 Index. Furthermore, the criterion validity was also confirmed through its significant inverse correlation with the U5MR, demonstrating its efficacy as a health determinant. Beyond statistical validation, these strong correlations simultaneously verify the critical relevance of the food security system to child health outcomes and emphasize the critical role of food security as an upstream determinant of child health, suggesting that improving health outcomes requires breaking sectoral silos to foster deep synergy between food security and public health interventions. Third, the CCD results indicate that the coordination among food security dimensions is generally limited worldwide. A consistent pattern where coupling degrees substantially exceed coordination levels was observed across all studied regions, especially in Sub-Saharan Africa. Despite high-dimensional coupling reflecting strong functional interdependence, this connectivity has not fostered effective synergy. Rather, it has resulted in a state of “low-level resonance”. In this state, the high interdependence of dimensions functions as a mechanism of mutual constraint rather than synergy. The deficiencies in one dimension can rapidly transmit risks throughout the entire system, weakening global resilience to climate- and health-related shocks. Consequently, negative feedback dynamics emerge, in which developmental lags across dimensions reinforce one another and neutralize the returns from isolated resource inputs. This may help explain why food insecurity often persists despite targeted development assistance and external support.

Our findings align with the findings in recent global assessments. Consistent with previous studies [16,30], our analysis confirms a distinct gradient and identifies the access and stability dimensions as the primary bottlenecks constraining overall food security performance in LMICs. This cross-study consistency suggests that the core pathology of global food insecurity has shifted from mere supply deficits to structural deficiencies in economic affordability and systemic resilience. However, regarding the construction of the indicator system, this study diverges methodologically from the previous literature, primarily in dimensional balance and indicator applicability. While Singh et al. innovatively introduced agency and sustainability dimensions, the lack of consistent, high-quality data for these metrics across LMICs limits their utility for cross-national comparison [31]. Conversely, Xu et al. simplified the assessment into three dimensions (quantity, economy, and resources). While computationally efficient, this approach explicitly excludes the utilization dimension [32]. Such production-centric, nutrition-neglecting selection may overlook the health consequences of hidden hunger, explaining why such indices often fail to establish strong correlations with health outcomes.

Regarding data selection strategies, although some previous research employed an extensive system of 34 indicators, their approach relies heavily on machine learning algorithms to impute widespread missing data [30]. While addressing data gaps, the reliance on synthetic data risks masking genuine extreme values of indicators related to stability (e.g., political instability) specific to fragile contexts. In contrast, the current study adopted 25 indicators with high data coverage in LMICs while adhering to the classic FAO four-dimension framework. We specifically prioritized direct measures within the utilization and stability dimensions. This indicator set, which is inclusive of health determinants and captures fewer, high-quality key variables, is more effective than broad but imputed datasets in reflecting the critical determinants of health within food systems. Beyond indicator selection, the application of the CCD model uncovers a structural reality obscured by linear assessments. Regions like Sub-Saharan Africa are trapped in a low-level resonance trap where developmental lags across dimensions are interlocked. This confirms that, consistent with the “One Health” framework, food systems function as highly entangled organic entities where deficits in any single dimension quickly propagate through tight coupling mechanisms, thereby compromising systemic resilience against climate and infectious threats.

The robust inverse correlation between the FSI and U5MR underscores the role of national food security as a foundational determinant of health system performance, operating through interconnected biological, socioeconomic, and systemic pathways. Biologically, adequate food availability and stability ensure maternal nutritional status and enhance pediatric immune function, directly reducing the incidence of adverse birth outcomes and susceptibility to infectious diseases such as pneumonia and diarrhea. Beyond these direct biological pathways, food security exerts an influence through socioeconomic channels. Improved agricultural stability often frees up household resources, enabling investments in healthcare, sanitation, and education. At the community level, stronger food systems are often linked to more robust public health infrastructure, including vaccination programs and maternal care services, which contribute to lower child mortality. Beyond direct health benefits, food security also reflects broader governance effectiveness, as it typically results from integrated policies connecting agriculture, social safety nets, and health systems. These coordinated efforts create synergistic outcomes, further reinforced by shared logistical infrastructure between food and health sectors, which facilitates the efficient delivery of medical interventions. Consequently, these findings highlight the importance of cross-sectoral approaches to child survival and population health, necessitating integrated policy frameworks that align agricultural interventions with public health objectives. Policy efforts should prioritize nutrition-sensitive social protection, align food security interventions with public health initiatives, and invest in climate-resilient agriculture. Such integration would not only advance progress toward SDG 2 but also accelerate the reduction in preventable under-five deaths and enhance overall health system performance, particularly in regions where food insecurity remains a critical challenge.

Our empirical findings also carry implications for reshaping global health governance. First, governance paradigms must shift from a yield-centric to a system-resilience approach. The CCD analysis reveals that while dimensional interdependence is universally high (C > 0.80), the stability dimension remains a critical global vulnerability. This indicates that traditional strategies focused solely on agricultural output are insufficient to secure public health in the climate crisis era. Global health governance requires adopting a whole-of-government strategy, dismantling barriers between the agricultural and health sectors. Climate-adaptive agriculture and supply chain diversification should be prioritized as upstream interventions to mitigate food system volatility and its cascading health crisis risks. Second, structural synergistic interventions within the “One Health” framework are imperative to address the low-level resonance trap. For regions like Sub-Saharan Africa, which are locked in a state of high coupling but low development, fragmented aid is ineffective. Policymakers should implement comprehensive intervention packages in which economic transfers to improve the access dimension are coordinated with investments in Water, Sanitation, and Hygiene (WASH) infrastructure to enhance utilization. Only through such concerted actions can positively feedback loops be triggered within the tightly coupled system, generating co-benefits for both food security and maternal and child health. Finally, multidimensional food security surveillance may be included in the early warning architecture of the global health security agenda. Given that the FSI demonstrates superior sensitivity as a health risk indicator compared to other composite indicators related to hunger or food systems, recognizing food insecurity as an early-warning indicator for public health emergencies will enable earlier identification of vulnerable populations, thereby strengthening the material foundation of universal health coverage by addressing inequities in food access or availability.

Although this study provides a novel integrated framework for assessing food security and its health implications in LMICs, several limitations should be acknowledged. First, the cross-sectional nature of the data limits the ability to establish causal relationships. While we demonstrated a robust inverse correlation between the FSI and the U5MR, this statistical correlation does not imply direct causation. Future research should utilize longitudinal panel data and advanced causal inference models to further explore the dynamic causal pathways between systemic food security transitions and population health outcomes. Second, the reliance on national-level aggregated data may mask subnational disparities. Food insecurity and child mortality are often characterized by significant internal heterogeneity within countries. National averages may mask localized pockets of acute food insecurity, as well as specific vulnerable groups in rural or conflict-affected areas. Future research should therefore adopt finer geographical resolution, integrating subnational- or household-level data to generate more precise evidence for targeted policy interventions. Third, while our 25-indicator system is comprehensive, it does not fully encompass all emerging dimensions of the One Health framework. Specifically, variables related to zoonotic disease risks, biodiversity loss, and the specific impact of climate extremes on food safety were not explicitly modeled. Future assessments can integrate more indicators to holistically evaluate the resilience of food systems against global health threats. Finally, the structural trap identified in this study, characterized by high coupling yet poor coordination, calls for further qualitative and context-specific investigation. While the CCD model has revealed the existence of such systemic constraints, the underlying socio-political and historical drivers in different countries remain to be examined. Comparative case studies of nations that have successfully escaped this trap could provide valuable practical insights for global health governance.

## 5. Conclusions

This study establishes a validated multidimensional assessment framework, confirming food security as a critical upstream determinant of child survival. The access and stability dimensions were identified as the primary lagging dimensions constraining systemic performance. The current food security system was characterized by high interdependence but insufficient synergy, and global governance must transcend traditional production-oriented paradigms to address these multidimensional structural imbalances and enhance systemic resilience.

## Data Availability

The data that support the findings of this study are available from the corresponding author, Minghui Ren, upon reasonable request. The analytical dataset was compiled and harmonized from multiple third-party sources, and its redistribution may be subject to source-specific terms and conditions.

## Supplementary Materials

The following supporting information can be downloaded at https://www.mdpi.com/article/10.3390/nu18091432/s1, Table S1: Weights of the indicators; Table S2: Single-Dimension Scores and Total Score of the Food Security in all Low- and Middle-Income Countries included in the study during 2019–2021.
