# Supplementary material for: A Multidimensional Assessment of Food Security in Low- and Middle-Income Countries: System Performance and Interdimensional Coordination
Source: Nutrients. 2026 Apr 30;18(9):1432. doi: 10.3390/nu18091432 (PMC13164969; doi:10.3390/nu18091432)
Supplement: Supplementary file 1 [file nutrients-18-01432-s001.zip › nutrients-4254115-supplementary.pdf]

Table S1. Weights of the indicators.

Variable Code	Indicator	Entropy	Weight 1	Weight 2
	**Availability**			
X101	Average dietary energy supply adequacy	0.996	0.046	0.011
X102	Share of dietary energy supply derived from cereals, roots and tubers	0.97	0.317	0.079
X103	Average protein supply	0.981	0.201	0.05
X104	Average supply of protein of animal origin (g/cap/day)	0.959	0.436	0.109
	**Access**			
X201	Rail lines density	0.815	0.573	0.143
X202	Gross domestic product per capita (in purchasing power equivalent)	0.918	0.254	0.063
X203	Prevalence of undernourishment	0.987	0.041	0.01
X204	Prevalence of severe food insecurity in the total population	0.988	0.038	0.01
X205	Prevalence of moderate or severe food insecurity in the total population	0.97	0.094	0.024
	**Utilization**			
X301	Percentage of children under 5 years of age who are stunted	0.982	0.095	0.024
X302	Percentage of children under 5 years of age affected by wasting	0.984	0.084	0.021
X303	Percentage of children under 5 years of age who are overweight	0.993	0.038	0.009
X304	Prevalence of obesity in the adult population (18 years and older)	0.983	0.093	0.023
X305	Prevalence of anemia among women of reproductive age (15-49 years)	0.979	0.11	0.027
X306	Prevalence of exclusive breastfeeding among infants 0-5 months of age	0.974	0.138	0.034
X307	People using safely managed drinking water services	0.989	0.057	0.014
X308	People using safely managed sanitation services	0.979	0.11	0.027
X309	People using at least basic drinking water services	0.984	0.084	0.021
X310	People using at least basic sanitation services	0.969	0.164	0.041
X311	Prevalence of low birthweight	0.995	0.028	0.007
	**Stability**			
X401	Per capita food supply variability	0.994	0.026	0.007
X402	Political stability and absence of violence/terrorism	0.979	0.098	0.024
X403	Value of food imports over total merchandise exports	0.995	0.025	0.006
X404	Percent of arable land equipped for irrigation	0.877	0.583	0.146
X405	Cereals imports dependency ratio	0.943	0.269	0.067

Notes: Weight 2 is the weight among the whole food security indicator system. Weight 2= Weight 1*0.25

Table S2. Single-Dimension Scores and Total Score of the Food Security in all Low- and Middle-Income Countries included in the study during 2019–2021.

Region	Country	Availability	Access	Utilization	Stability	Total
East Asia & Pacific	Cambodia	0.454	0.130	0.605	0.330	0.380
	China	0.557	0.356	0.791	0.834	0.634
	Democratic People's Republic of Korea	–	–	0.756	0.811	0.392
	Fiji	0.388	0.162	0.716	0.114	0.345
	Indonesia	0.405	0.197	0.672	0.566	0.460
	Kiribati	0.404	0.103	0.518	0.105	0.283
	Laos	0.442	0.130	0.580	0.375	0.382
	Malaysia	0.485	0.320	0.641	0.216	0.416
	Maldives	–	–	0.659	–	0.165
	Marshall Islands	–	–	0.696	–	0.174
	Micronesia (Federated States of)	0.424	–	0.681	–	0.276
	Mongolia	0.647	0.198	0.687	0.175	0.427
	Myanmar	0.437	0.208	0.585	0.576	0.451
	Papua New Guinea	0.340	0.073	0.372	0.617	0.350
	Philippines	0.437	0.135	0.695	0.785	0.513
	Samoa	0.559	0.134	0.787	0.107	0.397
	Solomon Islands	0.384	–	0.525	–	0.227
	Thailand	0.344	0.302	0.671	0.869	0.546
	Timor–Leste	0.378	0.099	0.532	0.430	0.360
	Tonga	–	–	0.701	–	0.175
	Vanuatu	0.350	0.123	0.528	0.104	0.276
	Viet Nam	0.493	0.285	0.668	0.820	0.567
Europe & Central Asia	Albania	0.537	0.388	0.667	0.763	0.589
	Armenia	0.517	0.576	0.722	0.702	0.629
	Azerbaijan	0.453	0.614	0.633	0.783	0.621
	Belarus	0.563	–	0.679	0.219	0.365
	Bosnia and Herzegovina	0.438	0.506	0.668	0.168	0.445
	Bulgaria	0.440	0.873	0.697	0.396	0.601
	Georgia	0.440	0.501	0.682	0.259	0.471
	Kazakhstan	0.533	0.352	0.676	0.358	0.480
	Kyrgyzstan	0.447	0.160	0.740	0.744	0.523
	Montenegro	0.592	0.497	0.654	0.091	0.459
	North Macedonia	0.383	0.648	0.678	0.728	0.609
	Republic of Moldova	0.424	0.738	0.674	0.955	0.698
	Serbia	0.599	0.839	0.669	0.345	0.613
	Tajikistan	0.382	0.155	0.671	0.744	0.488
	Türkiye	0.472	–	0.661	0.769	0.476
	Turkmenistan	0.516	–	0.694	0.646	0.464
	Ukraine	0.469	0.782	0.657	0.646	0.638
	Uzbekistan	0.502	0.280	0.605	0.792	0.545
Latin America & Caribbean	Argentina	0.585	0.297	0.755	0.366	0.500
	Belize	0.343	0.141	0.650	0.201	0.334
	Bolivia (Plurinational State of)	0.450	–	0.696	0.216	0.341
	Brazil	0.522	0.222	0.664	0.316	0.431

	Colombia	0.405	0.187	0.735	0.183	0.377
	Costa Rica	0.446	0.290	0.723	0.436	0.474
	Cuba	0.445	–	0.689	0.702	0.459
	Dominica	0.459	0.168	0.640	0.117	0.346
	Dominican Republic	0.351	0.202	0.628	0.736	0.479
	Ecuador	0.368	0.200	0.733	0.774	0.518
	El Salvador	0.408	0.585	0.745	0.347	0.521
	Grenada	–	–	0.673	–	0.168
	Guatemala	0.332	0.232	0.648	0.461	0.418
	Haiti	0.276	0.031	0.469	0.482	0.315
	Honduras	0.281	0.098	0.700	0.163	0.310
	Jamaica	0.443	0.121	0.659	0.391	0.404
	Mexico	0.523	0.396	0.688	0.564	0.543
	Nicaragua	0.353	–	0.663	0.276	0.323
	Paraguay	0.410	0.183	0.699	0.337	0.408
	Peru	0.473	0.161	0.752	0.321	0.426
	Russia	0.570	0.362	0.674	0.306	0.478
	Saint Lucia	–	–	0.615	–	0.154
	Saint Vincent and the Grenadines	0.524	0.175	0.659	0.215	0.393
	Suriname	0.390	0.204	0.613	0.270	0.369
Middle East & North Africa	Algeria	0.386	0.179	0.644	0.118	0.332
	Djibouti	0.309	0.700	0.473	0.077	0.390
	Egypt	0.467	0.222	0.684	0.582	0.489
	Iran	0.370	0.212	0.716	0.741	0.510
	Iraq	0.350	–	0.655	0.752	0.439
	Jordan	0.306	–	0.641	0.187	0.283
	Lebanon	0.337	0.767	0.671	0.698	0.618
	Libya	0.437	0.161	0.671	0.062	0.333
	Morocco	0.463	–	0.664	0.598	0.431
	Syrian Arab Republic	0.331	–	0.649	0.769	0.437
	Tunisia	0.407	0.300	0.634	0.461	0.450
	Yemen	0.335	0.069	0.416	0.190	0.252
South Asia	Afghanistan	0.384	0.057	0.554	0.679	0.419
	Bangladesh	0.425	0.451	0.590	0.816	0.570
	Bhutan	–	–	0.613	–	0.153
	India	0.322	–	0.566	0.859	0.437
	Nepal	0.391	0.108	0.655	0.808	0.491
	Pakistan	0.350	0.251	0.543	0.853	0.499
	Sri Lanka	0.374	0.571	0.748	0.811	0.626
Sub–Saharan Africa	Angola	0.345	0.101	0.493	0.153	0.273
	Benin	0.368	0.171	0.345	0.170	0.263
	Botswana	0.341	0.169	0.612	0.120	0.311
	Burkina Faso	0.387	0.110	0.359	0.198	0.264
	Burundi	–	–	0.516	–	–
	Cabo Verde	0.389	0.120	0.643	0.163	0.329
	Cameroon	0.333	0.100	0.459	0.159	0.263
	Central African Republic	0.265	0.037	0.371	0.173	0.212

	Chad	0.415	0.041	0.278	0.211	0.236
	Comoros	0.336	0.062	0.428	0.101	0.232
	Congo	0.385	0.089	0.426	0.084	0.246
	Côte d'Ivoire	0.408	0.124	0.422	0.141	0.274
	Democratic Republic of the Congo	0.398	0.053	0.404	0.179	0.259
	Equatorial Guinea	–	–	0.501	–	–
	Eritrea	–	–	0.411	–	–
	Eswatini	0.332	0.409	–	0.427	–
	Ethiopia	0.407	0.076	0.424	0.241	0.287
	Gabon	0.486	–	0.477	0.087	0.263
	Gambia	0.306	0.072	0.510	0.116	0.251
	Ghana	0.400	0.175	0.479	0.223	0.320
	Guinea	0.357	–	0.400	0.187	–
	Guinea–Bissau	0.334	0.069	0.429	0.222	0.263
	Kenya	0.288	0.134	0.486	0.144	0.263
	Lesotho	0.376	0.064	0.550	0.076	0.266
	Liberia	0.388	0.077	0.406	0.102	0.243
	Madagascar	0.398	0.072	0.372	0.344	0.297
	Malawi	0.392	0.157	0.499	0.276	0.331
	Mali	0.418	0.126	0.452	0.216	0.303
	Mauritania	0.434	0.112	0.491	0.119	0.289
	Mauritius	0.470	0.230	0.707	0.695	0.526
	Mozambique	0.370	0.098	0.411	0.130	0.252
	Namibia	0.344	0.120	0.523	0.109	0.274
	Niger	0.338	0.089	0.289	0.184	0.225
	Nigeria	0.344	0.123	0.409	0.202	0.270
	Rwanda	0.275	–	0.698	0.174	–
	Sao Tome and Principe	0.276	0.093	–	0.705	–
	Senegal	0.352	0.192	0.538	0.176	0.315
	Sierra Leone	0.383	0.076	0.408	0.182	0.262
	Somalia	0.209	0.027	0.363	0.063	0.166
	South Africa	0.434	0.436	0.589	0.302	0.440
	South Sudan	0.356	0.038	0.322	–	–
	Sudan	0.317	0.096	0.437	0.223	0.268
	Togo	0.361	0.213	0.415	0.181	0.293
	Uganda	0.189	0.057	0.497	0.202	0.236
	Tanzania	0.306	0.098	0.448	0.238	0.273
	Zambia	0.392	0.066	0.480	0.234	0.293
	Zimbabwe	0.354	0.187	0.506	0.165	0.303

Notes: The dash ("–") indicates missing scores for the single dimension or the total score. These scores were not calculated because data were unavailable for one or more indicators within that dimension, rendering the calculation impossible.
